# Novel PCR Assays Complement Laser Biosensor-Based Method and Facilitate *Listeria* Species Detection from Food

**DOI:** 10.3390/s150922672

**Published:** 2015-09-08

**Authors:** Kwang-Pyo Kim, Atul K. Singh, Xingjian Bai, Lena Leprun, Arun K. Bhunia

**Affiliations:** 1 Molecular Food Microbiology Laboratory, Department of Food Science, Purdue University, West Lafayette, IN 47907, USA; E-Mails: kpkim@jbnu.ac.kr (K.-P.K.); aksingh@purdue.edu (A.K.S.); bai16@purdue.edu (X.B.); l.leprun@laposte.net (L.L.); 2 Department of Food Science and Technology, College of Agriculture and Life Sciences, Chonbuk National University, Jeonbuk 561756, Korea; 3 Department of Comparative Pathobiology, Purdue University, West Lafayette, IN 47907, USA

**Keywords:** alcohol acetaldehyde dehydrogenase, *Listeria* adhesion protein, *Listeria* species, PCR, detection, BARDOT

## Abstract

The goal of this study was to develop the *Listeria* species-specific PCR assays based on a house-keeping gene (*lmo1634*) encoding alcohol acetaldehyde dehydrogenase (Aad), previously designated as *Listeria* adhesion protein (LAP), and compare results with a label-free light scattering sensor, BARDOT (bacterial rapid detection using optical scattering technology). PCR primer sets targeting the *lap* genes from the species of *Listeria sensu stricto* were designed and tested with 47 *Listeria* and 8 non-*Listeria* strains. The resulting PCR primer sets detected either all species of *Listeria* *sensu stricto* or individual *L. innocua*, *L. ivanovii* and *L. seeligeri*, *L. welshimeri*, and *L. marthii* without producing any amplified products from other bacteria tested. The PCR assays with *Listeria* *sensu* *stricto*-specific primers also successfully detected all species of *Listeria sensu stricto* and/or *Listeria innocua* from mixed culture-inoculated food samples, and each bacterium in food was verified by using the light scattering sensor that generated unique scatter signature for each species of *Listeria* tested. The PCR assays based on the house-keeping gene *aad* (*lap*) can be used for detection of either all species of *Listeria* *sensu stricto* or certain individual *Listeria* species in a mixture from food with a detection limit of about 10^4^ CFU/mL.

## 1. Introduction

*Listeria monocytogenes*, a foodborne pathogen, causes fatal systemic infection in immunocompromised hosts including the elderly, infants, pregnant women and their fetuses, HIV infected patients, and patients with malignancy receiving chemotherapy. Alcohol acetaldehyde dehydrogenase (Aad) in *L. monocytogenes* is a house-keeping enzyme and is involved in bacterial adhesion and paracellular translocation through epithelial barrier during intestinal phase of listeriosis [1,2,3,4]. Such a housekeeping enzyme with moonlighting function in prokaryotes plays an important role in pathogenesis [5,6]. The Aad (Lmo1634) is also known as *Listeria* adhesion protein (LAP) and its homolog is present in all species of *Listeria sensu stricto* (*i.e.*, in the narrow or strict sense) also known as archetypal *Listeria* species (*L. monocytogenes*, *L. ivanovii*, *L. seeligeri*, *L. welshimeri*, *L. innocua*, and *L. marthii*) [1,7,8]. Whereas, *L. floridensis*, *L. aquatic*, *L. cornellensis*, *L. riparia*, *L. grandensis*, *L. booriae*, *L. rocourtiae*, *L. newyorkensis*, *L. weihenstephanensis*, *L. fleischmannii* and *L. grayi* [8,9] are considered atypical (*sensu lato*: in the broad sense) and these group are phylogenetically divergent from the species of *Listeria* *sensu stricto* [9,10,11,12].

*L. monocytogenes* is pathogenic to humans and is responsible for fatal outbreaks involving ready-to-eat meat, dairy, fish, fruits, and vegetables [13]. It was responsible for 57 cases (22 fatalities) from consumption of tainted meat products in Canada [14], 27 cases (8 fatalities) from Quargel sour milk curd cheese [15], 147 cases (33 fatalities) from cantaloupe [16], and most recently in 2015, 35 cases (7 deaths) from caramel apple [17] and 10 cases (3 deaths) from ice cream [18]. The case-fatality rate for listeriosis is 20%–30% [19]. Under the United States Food and Drug Administration (FDA) definition of Current Good Manufacturing Practice, cGMP [21 CFR 110.5(a)], it is mandatory to monitor food for adulterations [21 U.S.C 342(a)] including all poisonous or deleterious substances, which may render food injurious to health. The FDA recommends initial rapid screening of frozen or refrigerated ready-to-eat (RTE) food products for *Listeria* species rather than the lengthy specific test for *L. monocytogenes* [20].

In this study, species of *Listeria* *sensu stricto*-specific PCR primer sets targeting *lap* (*aad*), a house-keeping gene were developed that detected all species of *Listeria* *sensu stricto* tested. House-keeping genes are integral and essential for bacterial metabolic function and survival [21], thus they provide an attractive target for detection. This molecular assay based on *lap* could be used as a screening tool to address the needs of food safety and the regulatory agency. These PCR primer sets were further used to detect *Listeria* species from inoculated food samples. In addition, the light scattering sensor, BARDOT (bacterial rapid detection using optical scattering technology) [22,23,24] was also employed to verify the presence of *L. monocytogenes* and *L. innocua* from a mixed culture (*Listeria* plus *Lactobacillus casei* and *Escherichia coli* O157:H7) inoculated food samples. In BARDOT, a red-diode laser (635 nm; 1 mW; 1 mm diameter) passes through the center of a bacterial colony on an agar plate and generates a 2-dimensional forward scatter fingerprint of each colony within 3–5 s [23]. Organism-specific features are extracted from scatter patterns and are used to identify unknown bacteria using the scatter image library [25]. Scatter image libraries for the thirteen serotypes of *L. monocytogenes* (1/2a, 1/2b, 1/2c, 3a, 3b, 3a, 4a, 4b, 4ab, 4c, 4d, 4e and 7) were also developed for the BARDOT-based detection in future studies.

## 2. Experimental Section

### 2.1. Bacterial Cultures, Growth and Ribotyping

All bacterial cultures (Table S1) used in this study are from our collection. All cultures were stored at −80 °C as 10% frozen glycerol stocks, and fresh cultures were obtained by propagating in Brain Heart Infusion broth (BHI) or Tryptic soy broth with 0.6% yeast extract (TSB-YE) at 37 °C for 16–18 h, with the exception of *L. rocourtiae*, which was grown at 32 °C. The bacterial cultures were plated on Brain Heart Infusion Agar (BHIA), Luria-Bertani Agar (LBA) to capture the colony scatter patterns. The majority of ribopattern information for cultures was obtained from a previous study from our lab [26]. Additional cultures were ribotyped using the automated Riboprinter^®^ (Qualicon, Inc., Wilmington, DE, USA) as described in our previousstudy [27]. For the food sample study, Fraser Broth (FB) containing 10 mL antimicrobial supplement (25 mg acriflavin, 20 mg nalidixic acid and 500 mg ammonium ferric citrate per liter) was used. Dehydrated media or media components were purchased from BD (Sparks, MD, USA) and FB was purchased from Acumedia (Neogen, Lansing, MI, USA).

### 2.2. Design of Lap Gene-Specific Primer Sets for Listeria Species

**Figure 1:**
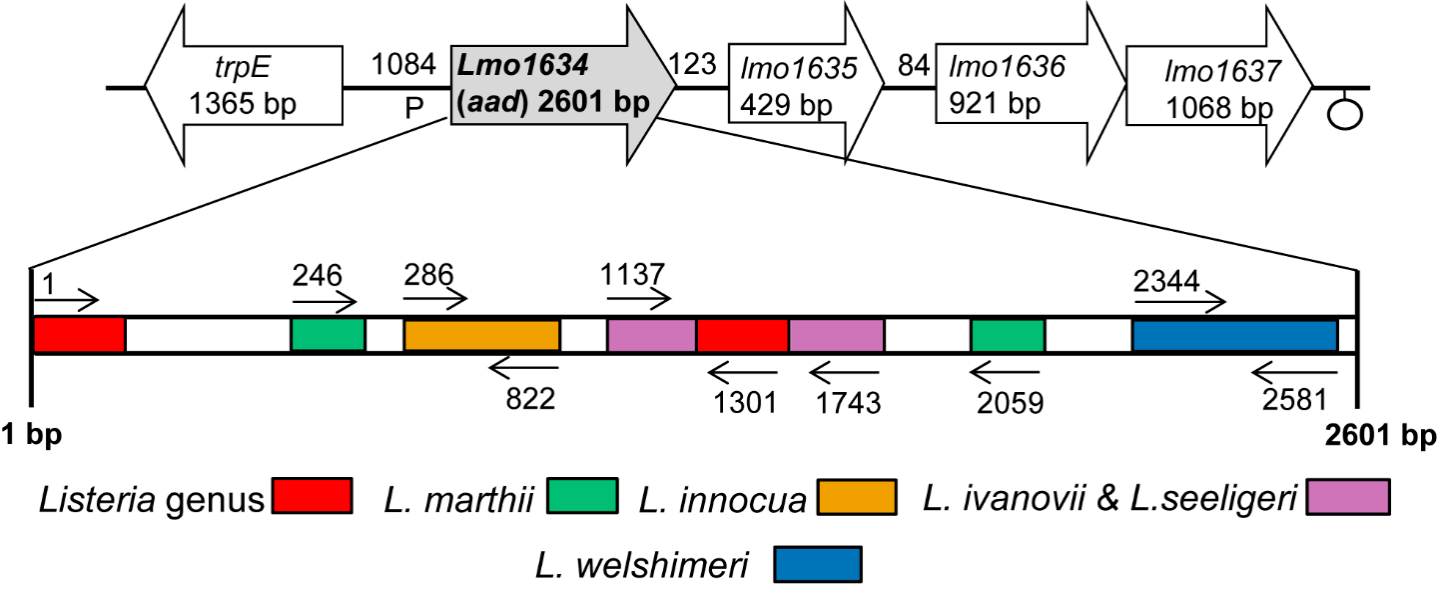
Schematic representation of the *lap* (*aad*) gene-specific primer binding sites for PCR-based detection of *Listeria*. Open block arrow represents genes flanking the *lap*. Abbreviations represents (similar proteins to): *trpE*, anthranilate synthase alpha subunit; *lmo1634*, alcohol-acetaldehyde dehydrogenase (LAP); *lmo1635*, unknown protein; *lmo1636*, ABC transporter (ATP-binding protein); *lmo1637*, membrane protein. The *Listeria* *lap* primers were specific for all species of *Listeria* *sensu stricto* tested, but not the atypical listeriae; *L. grayi* and *L. rocourtiae*. Colored boxes indicate primer binding sites for *Listeria* species (see Table 1 for PCR product size).

The *lap* sequences in *L. monocytogenes* F4244 (Acc. No. AY561824), *L. innocua* F4248 (Acc. No. AY561825), *L. welshimeri* ATCC35897 (Acc. No. AY561828), *L. seeligeri* SE31 (Acc. No. AY561827) and *L. ivanovii* SE98 (Acc. No. AY561826) were reported previously [1]. In addition, the complete sequences of the *lap* gene from *L. monocytogenes* EGD (Acc. No. NC_003210), *L. innocua* CLIP11262 (Acc. No. NC003212) and *L. marthii* (Acc. No. NZ_CM001047) [28] were obtained from NCBI GenBank [29]. To identify a species-specific DNA sequence region, the MultAlin [30] program was used to align and compare the sequences of the *lap* gene. The scheme for *Listeria* genus/species-specific primer binding sites on the *lap* gene are represented in Figure 1.

Two conserved sequence regions (1–54 and 1294–1401) were found in different *Listeria* species (*L. monocytogenes* EGD, F4244; *L. innocua* CLIP11262, F4248; *L. welshimeri*; *L. ivanovii*; *L. seeligeri* and *L. marthii*), and these regions were used to design the species of *Listeria sensu stricto*-specific primer set, ELAP-F2 and LIS-R1 (Table 1). Other *Listeria* species-specific primer sets were developed based on the rule that the 3′-end of primer should be unique to the target species. *L. innocua*-specific primers are designated as Inn-F1 and Inn-R1, and *L. welshimeri*-specific primers are named as Wel-F1 and Wel-R1. A primer set IvaSee-F1 and IvaSee-R1 was specific for both *L. ivanovii* and *L. seeligeri* as they represent close genetic relatedness [31]. In addition, primers Mar-F1 and Mar-R3 were specific for *L. marthii*. Specific primers for either *L. monocytogenes*, *L. ivanovii* or *L. seeligeri* could not be obtained, possibly due to their highly conserved *lap* gene sequence motifs [1].

**Table 1:** Sequences of species-specific primers based on *lap* sequence used in this study.

Primer	Sequence *^a^*	Location in *lap* Gene	Product Size (bp)	Specificity
ELAP-F1	5′CGGTCCCCGGGTACCATGGCAA	1–1301	1301	*Listeria* spp. (except *L. grayi*, *L. rocourtiae*)
LIS-R1	5′TTAAAGAAAATGCGGCC3′			
Inn-F1	5′GGAGTTATTAACGAAGATACT3′	286–822	536	*L. innocua*
Inn-R1	5′TTCTGCTTTTACTTCTTTAGCA3′			
IvaSee-F1	5′AAGCTGCAGTTATTCATTCC3′	1137–1743	606	*L. ivanovii*,*L. seeligeri*
IvaSee-R1	5′ATCTAAGAATTTTTGTTTTAGT3′			
Wel-F1	5′TTCTCGTATTATCGGTTTACCA3′	2344–2581	237	*L. welshimeri*
Wel-R1	5′GCTTCAAGATAGATTTCTTTCAA3′			
Mar-F1	5′AGAATATATTTGGAACAGCATC3′	246–268	1813	*L. marthii*
Mar-R1	5′GTTCGATTGCACGGATGGAAAG3′	2038–2059		

*^a^* Underlined indicates artificial nucleotide addition sites; translation start codon is indicated in bold character.

### 2.3. PCR Conditions, Primers and DNA Extraction

For PCR, 100 ng of template DNA, 25 pmol of each primer, 0.2 μL of Go*Taq* polymerase (5 U/μL stock; Promega), 1× Go*Taq* flexi colored buffer (5x stock, Promega), 2 mmol/L MgCl_2_ (25 mmol/L stock, Promega), and 200 µM of dNTPs (10 mmol/L stock, Promega) were mixed for a 25 µL final volume. PCR amplification was done using a thermocycler (GeneAmp PCR System 9700, Applied Biosystems) as follows: Hot start at 95 °C for 5 min; 30 cycles with denaturation at 95 °C for 1 min, annealing at 54 °C for 1 min, and extension at 72 °C for 1.5 min; final extension at 72 °C for 10 min. The amplified DNA was resolved in 1.2% agarose gel and visualized by ethidium bromide staining with a ChemiDoc XRS gel documentation system (Bio-Rad). The species of *Listeria* *sensu stricto*-specific primer set and individual *Listeria* species-specific primer sets (Table 1) were used for identification of each *Listeria* species in a pure culture and in the model foods. List of primers and their binding locations on *lap* gene is presented in Table 1. To further verify the *lap*-gene specific PCR results, two sets of cell wall hydrolase; CWH or p60 (*iap*) gene-specific primers, Lis1B/MonoA and Lis1B/Ino2 [32], were applied to verify *L. monocytogenes* and *L. innocua* cultures, respectively. The sequence of Lis1B, MonoA and Ino2 primers are 5′-TTATACGCGACCGAAGCCAAC-3′, 5′-CAAACTGCTAACACAGCTACT-3′ and 5′-ACTAGCACTCCAGTTGTTAAAC-3′, respectively.

The genomic DNA from reference cultures or enriched food samples were extracted with DNeasy Tissue Kit (Qiagen) following manufacturer’s protocol. Briefly, the cultures were pretreated with lysozyme solution (10 mg/mL in TE buffer (pH 7.0) containing 10 mmol/L Tris-Cl, pH 7.0 and 1 mmol/L EDTA) at 37 °C for 30 min prior to cell lysis. The total DNA was also extracted following the published protocol [33]. The concentration and purity of genomic DNA was determined using NanoDrop 2000C (Thermo Scientific, Franklin, MA, USA).

### 2.4. Specificity and Sensitivity of Lap Gene Primers for Listeria Detection

A total of 55 *Listeria* (*n* = 47) and non-*Listeria* (*n* = 8) cultures were tested to determine the specificity of *lap* gene primer sets for the species of *Listeria* *sensu stricto* or individual species: *L. monocytogenes* (*n* = 13), *L. ivanovii* (*n* = 12), *L. innocua* (*n* = 10), *L. seeligeri* (*n* = 5), *L. welshimeri* (*n* = 3), *L. grayi* (*n* = 2), *L. marthii* (*n* = 1) and *L. rocourtiae* (*n* = 1). Non-*Listeria* cultures included *Enterobacter aerogenes*, *Serratia marcescens*, *Hafnia alvei*, *Lactobacillus casei*, *Lactobacillus acidophilus*, *Bacillus cereus*, *Escherichia coli* O157:H7, and *Salmonella enterica* serovar Typhimurium (Table S1).

To determine the sensitivity (limit of detection) of the *lap* gene primer sets for detection of *Listeria* species, pure cultures of *L. monocytogenes* F4244 and *L. innocua* F4248 cells were plated on modified oxford (MOX) agar for enumeration. Simultaneously, total DNA was extracted in 200 µL of PBST (20 mM phosphate buffered saline (PBS), pH 7.2, with 0.05% Tween 20) from 1 mL of pure cultures using the boiling method [33]. A relationship between the number of cells and the corresponding DNA/genomic equivalents was established before performing the *lap* gene-based PCR to establish the sensitivity of the reaction. Overnight (16 h) grown cells of *L. monocytogenes* F4244 (8.02 ± 0.11 log_10_ CFU/mL) yielded 96.1 ± 2.1 ng/µL DNA, and 1 ng DNA was equivalent to 5.5 log_10_genomic equivalent (GE), whereas *L. innocua* F4248 (8.15 ± 0.24 log_10_ CFU/mL) yielded 98.4 ± 0.6 ng/µL DNA, and 1 ng DNA was equivalent to 5.5 log_10_ GE. PCR was performed with the diluted DNA and “GE was calculated. The genome size of *L. monocytogenes* and *L. innocua* 2.9 and 3.0 Mbp, “respectively, thus yielded 5.5 log_10_ GE for 1 ng of DNA. The amplified PCR products obtained from different cell concentrations were quantified using the NIH ImageJ tool, an image processing and analysis software [34].

### 2.5. Laser Optical Sensor and Scatter Image Analysis

Laser optical sensor, also designated BARDOT, works on the biophysical principles (refraction, diffraction, interference) of forward light scattering. An external design of BARDOT and its internal scheme has been described previously [23,35]. The detection time (sample-to-result) for the BARDOT-based detection of *Listeria* spp. colonies on BHI agar plate (BHIA) is about 22 h except for *L. rocourtiae*, which took about 48 h to generate the colony scatter pattern.

To find the optimal incubation time that generates maximal scatter features and distinguishing scatter patterns, a time-lapse study was performed to capture the scatter pattern of *Listeria* species at 17, 22 and 25 h. Scatter patterns were acquired for *Listeria* colonies after plating on BHIA, and each colony (~1 mm diameter) contained about 2.5 × 10^8^* Listeria* cells. The scatter patterns were captured when the colony size reached close to 1 mm in diameter. A total of 1,884 scatter images from pure cultures of eight *Listeria* species were captured on BHIA, where 677 scatter patterns were used to build the scatter image library and the rest of the scatter patterns were generated to find the optimal incubation time [24]. The scatter image library of eight *Listeria* species (*L. monocytogenes*, *L. innocua*, *L. grayi*, *L. seeligeri*, *L. welshimeri*, *L. marthii*, *L. ivanovii*, and *L. rocourtiae*) consisted of an average of 80 scatter patterns per species. This *Listeria* species library was used to differentiate *L. monocytogenes* and *L*. *innocua* inoculated in the food sample. Another scatter image library of *L. monocytogenes* and *L. innocua* (110 scatter images) was also built to specifically differentiate the two species. The scatter images were further processed and analyzed using a built-in image analysis software [25]. This analysis generated the cross validation matrix for the principal component analysis of the scatter images of the *Listeria* species. Scatter image libraries for 13 serovars of *L. monocytogenes* were also generated after growth on BHIA or LB agar (LBA) plates.

### 2.6. Detection and Identification of Listeria in Artificially Inoculated Food Samples

Two types of food samples, ready-to-eat hotdogs (franks) and cantaloupes, were procured from a local grocery store (West Lafayette, IN, USA). To test the application of designed primer sets, food samples were artificially inoculated and tested in three independent experimental replicates. Twenty-five grams of each hotdog and cantaloupe rinds (each piece was about 2 × 3 cm) were artificially inoculated with 100 CFU of single culture (*L. monocytogenes* F4244 or *L. innocua* F4248) or 100 CFU of a mixed culture (50 CFU of *L. monocytogenes* F4244 and 50 CFU of *L. innocua* F4248). Since we did not find any background microbial load in hotdogs, samples were inoculated with *Lactobacillus casei* (100 CFU/25g) and *Escherichia coli* (100 CFU/25g) as background contaminants. Four sets of food samples: (i) food alone; food inoculated with (ii) *L. monocytogenes*; (iii) *L. innocua*; and (iv) *L. monocytogenes* and *L. innocua*, were enriched in FB according to the USDA-FSIS protocol [36]. All inoculated food samples (25 ± 2 g) were enriched in 225 ± 2.5 mL of FB at 37 °C in a shaking incubator (140 rpm) for 24 h. One milliliter of enriched broth was used for the DNA extraction (as described before) and plated on MOX agar plates for enumeration and BHIA for identification by BARDOT [23,37]. Briefly, for BARDOT analysis, FB enriched samples were decimally diluted in 20 mmol/L PBS (pH 7.2), plated on BHIA, and incubated at 37 °C for 24 h or until the colony diameter reached 1.1 ± 0.2 mm. The scatter patterns of colonies were compared with the scatter pattern library of *Listeria* species for identification [23,24].

## 3. Results

### 3.1. Specificity and Sensitivity of Lap Gene Primers for Listeria Detection

**Figure 2:**
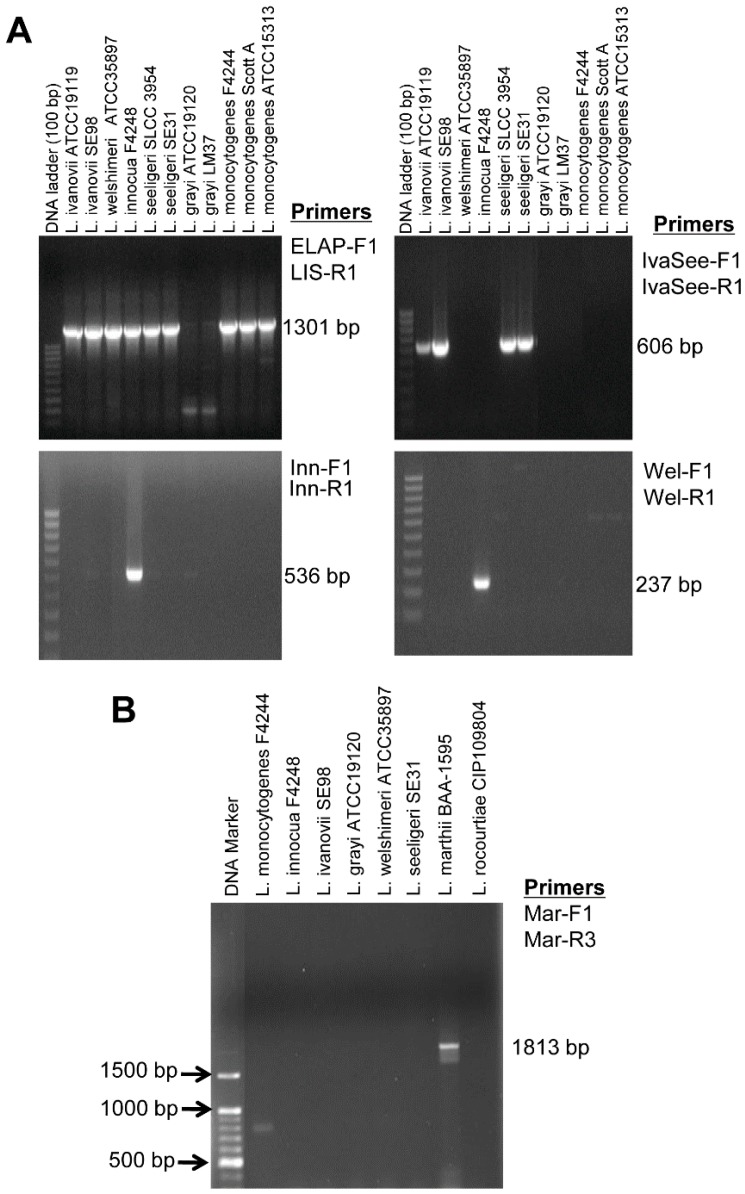
Representative agarose gel showing PCR amplification of selected *Listeria* species by using species-specific primers. (**A**) PCR results based on the species of *Listeria sensu stricto*-specific, and *L. innocua*-, *L. ivanovii*- and *L. seeligeri*- and *L. welshimeri*-specific primers. (**B**) PCR result using *L. marthii*-specific primers.

A total of 55 different *Listeria* (*n* = 47) and non-*Listeria* (*n* = 8) cultures were analyzed (Table S1). When the general *Listeria* primer set, ELAP-F2 and LIS-R1, was used, all the tested species of *Listeria* *sensu stricto* produced a 1301 bp band (Figure 2A, Table S1). When the *L. innocua*-specific primer set, Inn-F1 and Inn-R1, was used, a 536 bp band was amplified only in the *L. innocua* strains (Table S1) Likewise, the IvaSee-F1 and IvaSee-R1 primer set produced *L. ivanovii* and *L. seeligeri*-specific 606 bp band. The primer set, Wel-F1 and Wel-R1, generated 237 bp band only in the *L. welshimeri* strains, and a *L. marthii*-specific primer set, Mar-F1 and Mar-R3, produced a 1813 bp band without showing any amplified products from the other *Listeria* species (Figure 2B, Table S1).

These results demonstrated that the general *Listeria* primer set (ELAP-F2 and LIS-R1) could detect all the tested species of *Listeria* *sensu stricto*. The IvaSee-F1 and IvaSee-R1 were able to differentiate either *L. ivanovii* or *L. seeligeri* from other *Listeria* species or non-*Listeria* organisms without giving any false-positive results. *L. ivanovii* and *L. seeligeri* contain virulence gene sequences in their genome similar to *L. monocytogenes* [31], and exhibited high sequence homology in *lap* [1].

The *L. innocua*-specific primers (Inn-F1 and Inn-R1) were highly specific and did not give any PCR products with other *Listeria* species, including *L. monocytogenes* strains. This primer set could be used to detect *L. innocua* as a mixed culture with *L. monocytogenes.* Since these two species are usually found together in food and other ecological habitats, the presence of *L. innocua* could be used as an indicator for *L. monocytogenes* [38,39,40,41].

Use of the *L.* *welshimeri*-specific primer set, Wel-F1 and Wel-R1, successfully produced a PCR product in all four *L. welshimeri* strains tested. Since we could not design *L. monocytogenes*, *L. ivanovii* or *L. seeligeri*-specific primer sets, PCR assays for detection of these individual species were not possible with the primers sets used in this study.

Since 2010, eleven new *Listeria* species (a total of 17 species) were added to the genus *Listeria*; *L. marthii* [42], *L. rocourtiae* [11], *L. weihenstephanensis* [12], *L. fleischmannii* [10,43], *L. floridensis*, *L. aquatic*, *L. cornellensis*, *L. riparia*, *L. grandensis* [8], *L. booriae*, and *L. newyorkensis* [9]. The presence of a *lap* homologue in *L. marthii* [7] has been reported and the resulting primer set (Mar-F1 and Mar-R3) is specific, but we were unable to obtain any *lap* gene based primers for *L. rocourtiae*, *L. weihenstephanensis*, *L. fleischmannii* and *L. grayi*. These are considered atypical and are phylogenetically divergent from the species of *Listeria* *sensu stricto* within the genus *Listeria* [1,11,43]. *L. grayi* and *L. rocourtiae* did not give any amplification with the species of *Listeria* *sensu-stricto*-specific primer set indicating the possible sequence heterogeneity in the *lap* sequence in these atypical listeriae (Table S1).

The specificity of all primer sets was examined with eight non-*Listeria* cultures and none of them yielded any PCR product (Table S1). We were even able to identify four mislabeled microorganisms: Two with general *Listeria* primer set, one of each with *L. innocua*-specific and *L. welshimeri*-specific primer set (Table S1). Ribotyping identified them as *L. monocytogenes* DUP-1035 and DUP-1039, *L. welshimeri* DUP-1074 and *L. innocua* DUP-1009 (Table S1).

**Figure 3:**
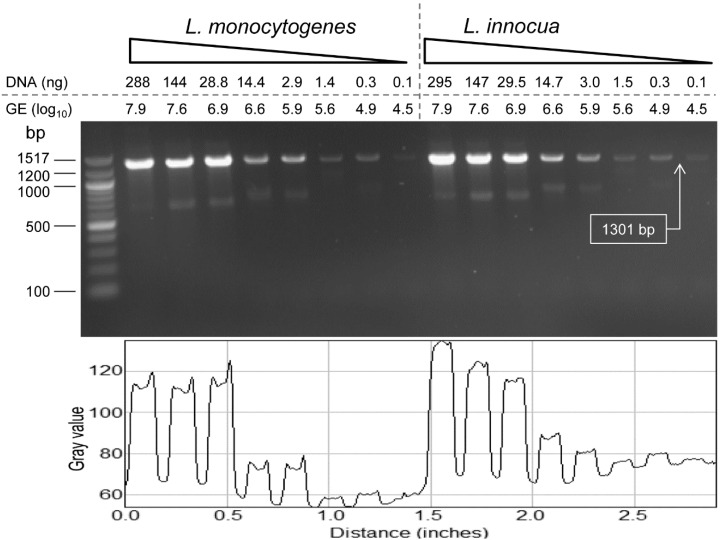
Sensitivity of *lap* gene-based *Listeria* species *sensu stricto*-specific primer (ELAP-F1/LIS-R1) tested against *L. monocytogenes* F4244 and *L. innocua* F4248. Agarose gel showing amplifications with the primers for different concentrations of template DNA of *L. monocytogenes* and *L. innocua* in reaction volume of 25 µL. PCR products in the gel were quantified using the NIH ImageJ image processing and analysis software.

The detection limit (sensitivity) of PCR with the species of *Listeria* *sensu stricto*-specific primer (ELAP-R1/LIS-R1) was 4.5 log_10_ genome equivalents for both *L. monocytogenes* and *L. innocua* (Figure 3). PCR for the DNA sensitivity was performed with the total DNA extracted from 1 mL culture of *L. monocytogenes* F4244 (8.02 ± 0.11 log_10_ CFU/mL) and *L. innocua* F4248 (8.15 ± 0.24 log_10_ CFU/mL) that also indirectly depicted the PCR sensitivity for the bacterial cell number. One milliliter cultures of *L. monocytogenes* and *L. innocua* yielded 96.1 ± 2.1 ng/µL and 98.4 ± 0.6 ng/µL of DNA concentrations, respectively. The genome equivalents were calculated from the genome size (*L. monocytogenes* size is 2.9 × 10^6^ bp and *L. innocua* is 3.0 × 10^6^ bp), and the molecular weight of nucleotide (1 bp = 650 Da).

### 3.2. Scatter Image Library of Listeria Species and Serovars

**Figure 4:**
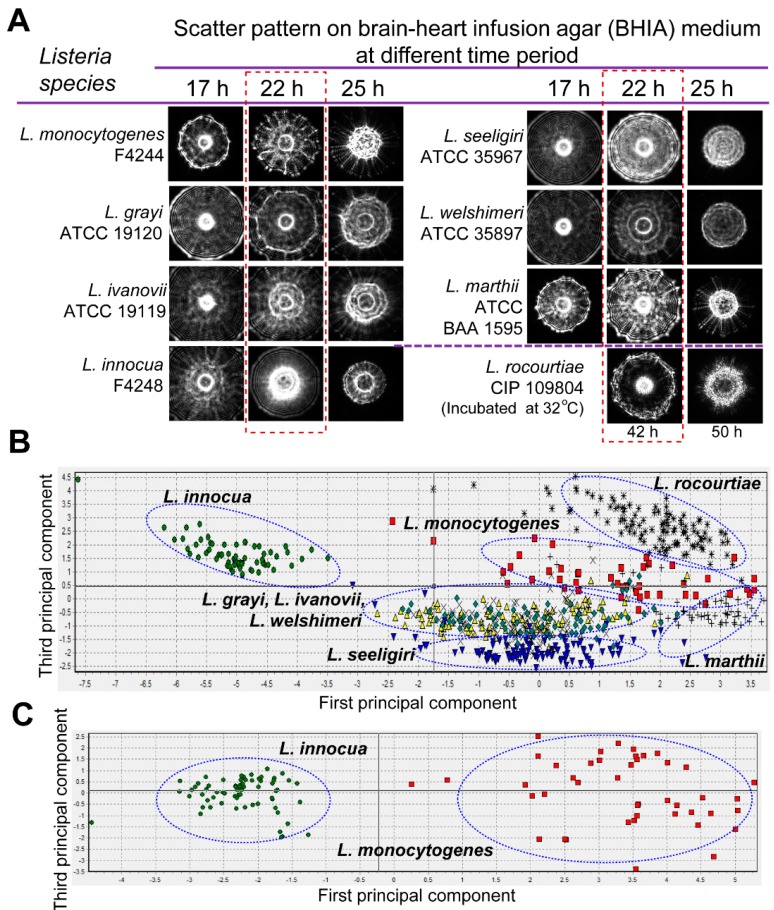
Optical scatter patterns of *Listeria* species and image analysis. (**A**) Colony scatter patterns were captured using BARDOT at different incubation times for eight *Listeria* species on BHI agar plates. Rectangular selection with broken line depicts the optimal incubation time (22 h) that yielded differentiating scatter images when the colony size was 1.1 ± 0.2 mm diameter; (**B**) Principal component analysis of the eight *Listeria* species used to build the scatter image library. Blue oval selections indicate grouping of the *Listeria* species; (**C**) Principal component analysis of *L. monocytogenes* and *L. innocua* colony scatter images that were used to build a two-species scatter image library. The blue oval selections indicate grouping of each *Listeria* species.

The light scattering sensor (BARDOT) generated distinguishing forward scattering patterns for colonies of *Listeria* species on BHIA. Time-lapse measurement of the scatter patterns indicated that *Listeria* species generated scatter patterns with maximal differential scatter features at 22 h of incubation (Figure 4A). Principal component analysis performed on the basis of cross validation matrix revealed that *L. innocua*, *L. rocourtiae*, *L. monocytogenes*, *L. marthii*, and *L. seeligeri* can be grouped separately based on the differences in the scatter patterns (Figure 4B) with 100%, 100%, 97.7%, 95.2% and 94.9%, positive predictive value (PPV), also known as classification accuracy, respectively (Table S2). However, *L. grayi*, *L. ivanovii*, and *L. welshimeri* could not be differentiated based on the scatter patterns on BHIA. Application of the *L. monocytogenes* and *L. innocua*-specific image libraries, generated even higher PPVs of 100% for both of the species, and they grouped separately in the principal component analysis (Figure 4C). These image libraries were also used to match scatter images of *L. monocytogenes* and *L. innocua* that were obtained from artificially inoculated food samples mentioned below in the result section.

**Figure 5:**
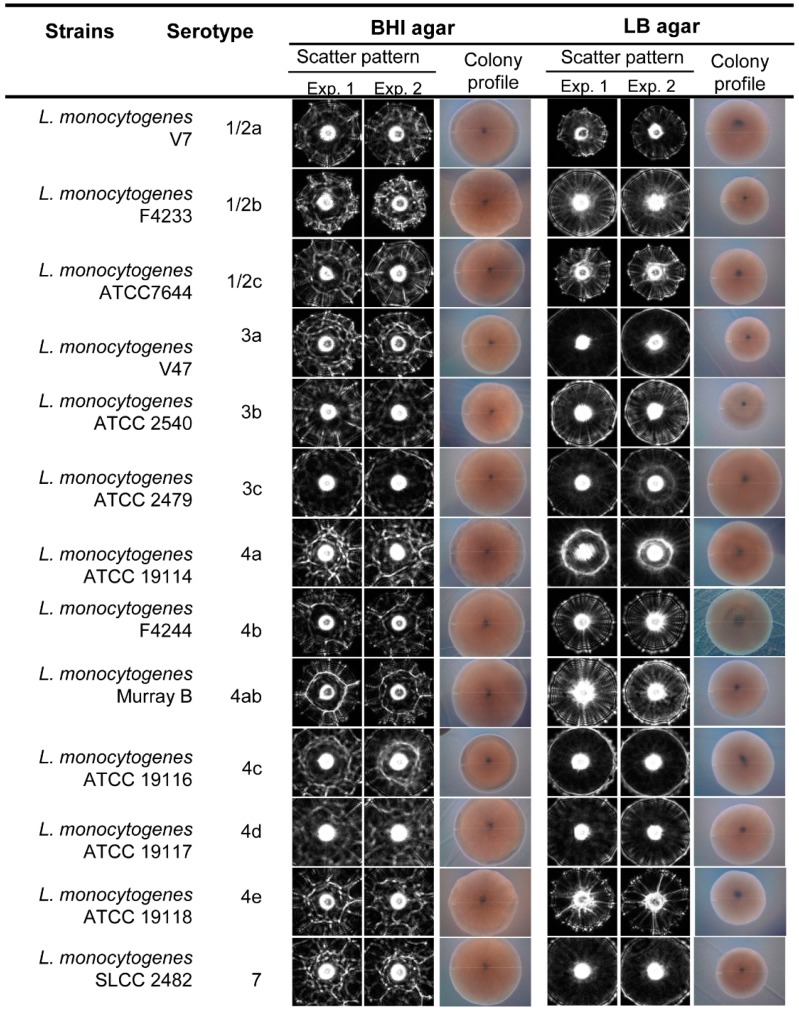
Representative scatter images of colonies of *L. monocytogenes* serotypes grown on BHI and LB agar. Between 50 and 100 colony scatter images for each serovar were collected from each experiment. Colony profiles were measured under phase contrast microscope with 10× objective when the colony size was 1.1 ± 0.2 mm diameter on BHI and LB agar after 21–23 h and 25–27 h of incubation, respectively.

In this study we also tested the capabilities of the laser sensor to differentiate *L. monocytogenes* at the serovar level after growth on BHI and LB agar plates (Figure 5). Differences at the serovar level were observed after analysis using the cross validation matrix, where a high PPV average was observed on LB agar compared to the BHI agar media, 90.1% and 82.9%, respectively (Table 2). Scatter pattern analysis for the thirteen serotypes underscores the feasible application of the laser optical sensor to generate a scatter image library with differentiating scatter patterns for *L. monocytogenes* serotypes that can be used for screening and detection of *L. monocytogenes* at the serovar level.

**Table 2:** Positive predictive value (PPV, precision rate) for the scatter images of colonies obtained from thirteen *L. monocytogenes* serotypes grown on BHIA and LBA media.

Strains	Serotype	% Average Positive Predictive Value (PPV ± SD)	
		BHI	LB
*L. monocytogenes* V7	1/2a	81.8 ± 2.3	96.2 ± 1.9
*L. monocytogenes* F4233	1/2b	89.4 ± 1.1	96.8 ± 2.1
*L. monocytogenes* ATCC7644	1/2c	74.5 ± 3.2	81.3 ± 3.8
*L. monocytogenes* V47	3a	80.8 ± 1.8	77.6 ± 5.6
*L. monocytogenes* ATCC 2540	3b	74.2 ± 2.2	97.6 ± 1.7
*L. monocytogenes* ATCC 2479	3c	99.8 ± 0.9	85.2 ± 2.3
*L. monocytogenes* ATCC 9114	4a	93.2 ± 1.3	98.0 ± 1.8
*L. monocytogenes* F4244	4b	46.2 ± 3.5	88.8 ± 2.1
*L. monocytogenes* Murray B	4ab	92.8 ± 2.9	85.4 ± 3.2
*L. monocytogenes* ATCC 19116	4c	98.8 ± 1.0	93.6 ± 2.8
*L. monocytogenes* ATCC 19117	4d	99.0 ± 0.5	88.2 ± 3.1
*L. monocytogenes* ATCC 19118	4e	65.4 ± 5.6	92.6 ± 1.7
*L. monocytogenes* SLCC 2482	7	82.4 ± 2.4	90.2 ± 3.1
Average precision rate		82.9 ± 2.2	90.1 ± 2.7

### 3.3. Detection and Verification of Listeria from Food Samples

**Figure 6:**
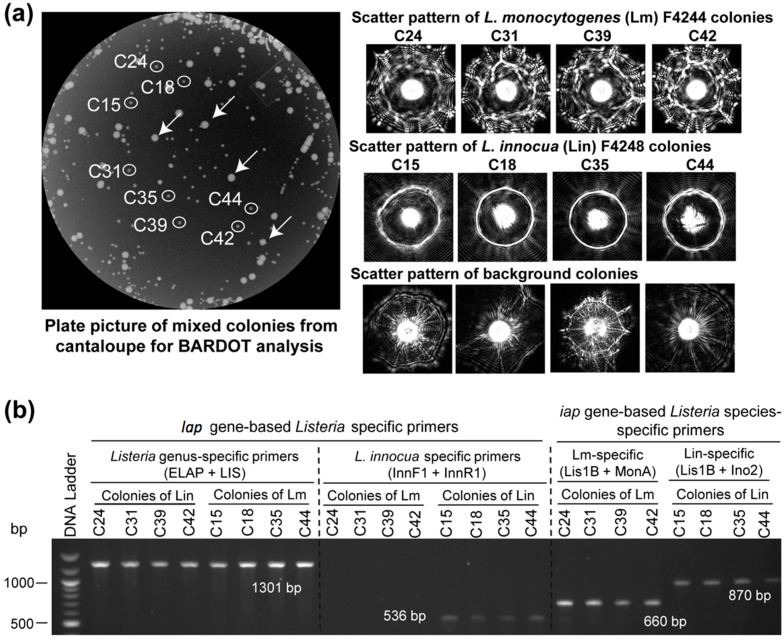
Detection and verification of *L. monocytogenes* (Lm) and *L. innocua* (Lin) in mixture from inoculated cantaloupe samples with BARDOT and PCR. (**A**) Enriched cantaloupe samples containing *L. monocytogenes* and *L. innocua* were plated on BHI agar and colony scatter patterns were obtained. Scatter patterns were matched against the BARDOT scatter image library for *Listeria* identification. The white arrows indicate background bacterial colonies from the cantaloupe; (**B**) BARDOT identified colonies were picked and tested with the primer sets specific for species of *Listeria* *sensu stricto* (ELAP-F1/LIS-R1), *L. monocytogenes* (Lis1B/MonA), and *L. inn*ocua (InnF1/InnR1, Lis1B/Ino2) for verification of colonies.

The ability of *lap* gene-specific primer sets to detect *L. monocytogenes* and *L. innocua* from inoculated food samples were verified (Figure S1, Table 3). Since a *lap* gene-based *L. monocytogenes* specific primer set could not be designed, we used the combination of species of *Listeria* *sensu stricto*-specific and *L. innocua* specific primer sets to detect *Listeria* from food. The food samples that revealed positive amplification with ELAP-F1 & LIS-R, but did not show any amplification with the *L. innocua*-specific primer sets (Inn-F1 & Inn-R1), were considered to contain any *Listeria* spp. other than the *L. innocua* (Table 3, Figure S1). Samples with positive amplification for both the primer sets (ELAP-F1 & LIS-R1 and with Inn-F1 & Inn-R1) corroborated the presence of *L. innocua* in the food sample (Table 3). Background microbial colonies obtained from the un-inoculated cantaloupe did not result in any positive amplification (Figure 6). This highlights the specificity and applicability of *lap* gene-specific primers for detection of *Listeria* species even in food samples with background microbiota. We further verified these results by analyzing the enriched food samples by BARDOT.

In our previous study, BARDOT generated distinct signature scatter patterns for the colonies of *L. monocytogenes* or *L. innocua* in mixed culture [23]. The distinctive scatter patterns generated with BARDOT facilitated accurate identification of *L. monocytogenes* or *L. innocua* or both in food samples after matching the scatter patterns with the respective image libraries (Figure 6). Colonies # C42, C41, C43, C34, and C29 originated from *L. monocytogenes* and *L. innocua*-inoculated hotdog sample on BHIA were identified as *L. innocua*, while colonies # C28, C25, C23, and C5 were identified as *L. monocytogenes* after comparing scatter images with the library (Figure 6A). These colonies were initially identified as *Listeria* spp. by PCR with the primer set (ELAP-F1/LIS-R1) designed in this study (Figure 6B). Further, these colonies were also confirmed at the species level (*L. monocytogenes* and *L. innocua*) using the primers for the *iap* gene [32,44]. The scatter patterns of *L. innocua*, when matched with the libraries of *Listeria* species as well as *L. monocytogenes* and *L. innocua*, generated 100% match with scatter image library. *L. monocytogenes* colonies from the artificially inoculated hotdog sample revealed low PPV (<80%) when matched with the libraries of *Listeria* species; however, the same *L. monocytogenes* colony scatter pattern generated a high PPV (>90%) when matched with the *L. monocytogenes* and *L. innocua* library. The low PPV of *L. monocytogenes* with the *Listeria* species library could be attributed to the overlapping pattern of *L. monocytogenes* with the scatter pattern of other *Listeria* species. BARDOT-based identification of *L. monocytogenes* and *L. innocua* colonies along with PCR analysis with the *lap* and *iap* gene-specific primers resulted in 100% and 100% identification, respectively, for both the *Listeria* species.

**Table 3:** *Listeria* detection using *lap* gene-specific primers in food systems.

Treatment *^a^*	Inoculation (CFU/25g)	Enrichment Time (h) in Fraser Broth at 37 °C	PCR *^b^*			
			Hotdog		Cantaloupe	
			ELAP-F1/LIS-R1	Inn-F1/Inn-R1	ELAP-F1/LIS-R1	Inn-F1/Inn-R1
Uninoculated	0	24	−	−	−	−
*L. innocua* (Lin)	100	24	+	+	+	+
*L. monocytogenes* (Lm)	100	24	+	−	+	−
Lin and Lm*^c^*	100	24	+	+	+	+
*Lb. casei*	100	24	−	−	−	−
*E. coli*	100	24	−	−	−	−

*^a^* Three independent experiments were performed for each food sample; *^b^* DNA extracted from broth enrichment following the published protocol [33] were amplified with the *lap* gene specific primers for species of *Listeria sensu stricto* (ELAP-F1/LIS-R1) and *L. innocua* (Inn-F1/InnR1); *^c^* Food samples were inoculated with 50 CFU each of *L. monocytogenes* (Lm) and *L. innocua* (Lin) in 25 g of food sample.

## 4. Discussion

This study reports the application feasibility of primer sets designed from a gene encoding the house-keeping alcohol acetaldehyde dehydrogenase (*aad*), also known as *Listeria* adhesion protein (*lap)*, for detection of *Listeria* at the genus and species level. This highly conserved house-keeping enzyme is involved in the pathogenesis of virulent *Listeria* but not avirulent *Listeria* species [1,45] thus providing an attractive target for *Listeria* detection. The *aad* (*lap*) sequence is conserved (97%–98% homology) in the species of *Listeria* *sensu stricto* and yielded a primer set (ELAP-F1 and LIS-R1) that detected these *Listeria* *sensu stricto* (archetypal) species, *-*but not the atypical (*Listeria sensu lato*) listeriae: *L. grayi* and *L. rocourtiae*. Even though both *L. grayi* and *L. rocourtiae* possess a *lap* (*aad*) homolog, they did not give any amplification with the species of *Listeria* *sensu stricto*-specific primer set indicating a possible sequence heterogeneity in the *lap* gene in these atypical listeriae. Indeed, *lap* gene sequence comparison between *L. monocytogenes* F4244 (AY561824) and *L. grayi* DSM 20601 (NZ_GL538352.1) or *L. rocourtiae* FSL F6-920 c6 (NZ_AODK01000006.1) revealed only 80% homology. The other newly isolated *Listeria* species were not tested in the PCR assay, but we anticipate negative results for these species since they are genetically similar to the two atypical listeriae tested in this study [8,9]. Furthermore, a minor variation in *lap* sequences (97%–98% similarity) among the different species of *Listeria* *sensu stricto* [1] yielded highly specific primer sets for *L. innocua*, *L. welshimeri*, *L. marthii*, and *L. ivanovii* and *L. seeligeri* together, but none for *L. monocytogenes* (Figure 1, Table S1). These could be useful for specific identification at the species level. *Listeria* *sensu stricto*-specific and other species-specific sets of primers also did not amplify any non-listerial bacteria tested with pure cultures or in a food matrix (Tables S1 and 3), highlighting the specificity of the *lap* gene-specific primer sets for *Listeria* spp. The detection limit of primer sets with the diluted template DNA revealed an indirect detection limit of about 4.5 log_10_ genome equivalents for this assay. These primer sets could be used to detect and identify *Listeria* species during screening of frozen or refrigerated ready-to-eat (RTE) food products as recommended by the FDA [20]. Application of PCR-based assay targeting gene encoding house-keeping enzyme (cell wall hydrolase; CWH or p60 encoded by *iap*) in *Listeria* spp. was reported earlier, in which species-specific primer sets successfully detected each *Listeria* spp.; *L. monocytogenes*, *L. ivanovii*, *L. innocua*, *L. seeligeri*, *L. welshimeri* within the genus, except *L. grayi* [32]. Similarly, PCR assay targeting genes encoding aminopeptidase and fibronectin-binding protein were also used for rapid detection of *L. monocytogenes* [46,47].

We developed the light scattering sensor, BARDOT, through collaborative efforts with engineers at the Center for Food Safety Engineering at Purdue University [48]. We have successfully used BARDOT to differentiate and detect *L. monocytogenes*, *L. ivanovii*, *L. innocua*, *L. seeligeri*, *L. welshimeri* and *L. grayi*. The BARDOT system was also successfully applied to differentiate *L. monocytogenes* from other pathogens (*Salmonella* *enterica* serovar Enteritidis and Typhimurium, *E. coli* O157:H7) based on the scatter patterns from artificially inoculated ready-to-eat hotdog, shredded beef, raw ground beef and chicken, frozen and fresh spinach, and fresh tomato [37]. Recently, we have also optimized the BARDOT-based method for detection and screening of several additional foodborne pathogens including *Bacillus* spp. [49], *Campylobacter* spp. [50], *Salmonella enterica* serovars [24], Shiga-toxigenic *E. coli* [35], and *Vibrio* spp. [51]. In this study, we used BARDOT to differentiate the species of *Listeria* when grown on BHIA. On BHIA, BARDOT successfully differentiated *L. monocytogenes*, *L. innocua*, *L. rocourtiae*, *L. marthii*, and *L. seeligeri*; however, it did not yield satisfactory differential patterns of *L. grayi*, *L. ivanovii*, and *L. welshimeri* (Figure 4B). In our previous report we have shown that the BHIA and modified Oxford agar prepared without ferric ammonium citrate were able to successfully differentiate the colonies of *L. monocytogenes*, *L. ivanovii*, *L. innocua*, *L. seeligeri*, *L. welshimeri* and *L. grayi*, and colonies of *L. monocytogenes* from *L. innocua*, respectively, based on scatter signature patterns [23,44]. These findings reaffirm the media-dependent generation of scatter signatures for bacterial identification.

*L. monocytogenes* is the primary human pathogen in the genus *Listeria* and among the 13 serotypes, serotype 1/2a and 4b are responsible for ~75% of all *L. monocytogenes* related outbreaks. Here we have shown that BARDOT can differentiate *L. monocytogenes* serovars 1/2a and 4b with high accuracy on LBA with 96.2% ± 1.9% and 88.8% ± 2.1% PPV, respectively. Observed differences in the scatter pattern of different serovars could be attributed to the O (somatic) antigens expressed on the surface of *L. monocytogenes* [52]. Furthermore, metabolic activity and genomic differences between different species or serotypes of *Listeria* can also contribute to the differential scatter patterns [37]. The genome size for serotype 1/2a (*L. monocytogenes* F6854) is 2.97 × 10^6^ bp, with a total of 3028 genes, of which 2963 are protein coding genes. Serotype 4b (*L. monocytogenes* F2365) has a genome size of 2.91 × 10^6^ bp, with a total of 2933 genes, of which 2848 are protein coding genes. Thus, a difference of 115 protein coding genes in serotype 1/2a to that of 4b could be crucial in generating differential scatter patterns for these two serotypes. In a comparative whole genome sequencing study, it was found that 83 genes were restricted to 1/2a serotype and 51 genes were restricted to 4b serotype [53].

## 5. Conclusions

In summary, *lap* gene based *Listeria* *sensu stricto* and individual species-specific primers successfully detected all tested species of *Listeria* *sensu stricto* (archetypal) while some limitations for individual species level detection. The PCR based assays with the species of *Listeria* *sensu stricto*-specific primer sets based on *lap* and *iap* genes also successfully detected *L. monocytogenes* and *L. innocua* from mixed culture-inoculated food samples, and each bacterium in food was verified by the light scattering sensor that generated unique scatter signature for each species of *Listeria*. These data emphasize that the BARDOT system could be used to identify *Listeria* spp. on agar plates from a mixed cultures and may serve as a complimentary tool when testing samples with nucleic acid-based molecular methods.

## Supplementary Files

Supplementary File 1
