# Erratum: Kim, K.-P.; Singh, A.K.; Bai, X.; Leprun, L.; Bhunia, A.K. Novel PCR Assays Complement Laser Biosensor-Based Method and Facilitate *Listeria* Species Detection from Food. *Sensors* 2015, *15*, 22672–22691

**DOI:** 10.3390/s17050945

**Published:** 2017-04-25

**Authors:** Kwang-Pyo Kim, Atul K. Singh, Xingjian Bai, Lena Leprun, Arun K. Bhunia

**Affiliations:** 1 Molecular Food Microbiology Laboratory, Department of Food Science, Purdue University, West Lafayette, IN 47907, USA; kpkim@jbnu.ac.kr (K.-P.K.); aksingh@purdue.edu (A.K.S.); bai16@purdue.edu (X.B.); l.leprun@laposte.net (L.L.); 2 Department of Food Science and Technology, College of Agriculture and Life Sciences, Chonbuk National University, Jeonbuk 561756, Korea; 3 Department of Comparative Pathobiology, Purdue University, West Lafayette, IN 47907, USA

The authors wish to correct the oligonucleotide sequence of *primer E-LAP-F1* and LIS-R1 in Table 1 in their paper published in *Sensors* [1], doi:10.3390/s150922672, http://www.mdpi.com/1424-8220/15/9/22672. The following table should be used.

The changes do not affect the scientific results. The manuscript will be updated and the original will remain online on the article webpage, with a reference to this Erratum.

## Figures and Tables

**Table 1:** Sequences of species-specific primers based on *lap* sequence used in this study.

Primer	Sequence *^a^*	Location in *Lap* Gene	Product Size (bp)	Specificity
ELAP-F1	5′CGGTCCCCGGGTACC**ATG**GCAATTAAAGAAAATGCGGCC3′	1–1301	1301	*Listeria* spp. (except *L. grayi*, *L. rocourtiae*)
LIS-R1	5′TTTGTGATACAGAGTTTTTACC3′			
Inn-F1	5′GGAGTTATTAACGAAGATACT3′	286–822	536	*L. innocua*
Inn-R1	5′TTCTGCTTTTACTTCTTTAGCA3′			
IvaSee-F1	5′AAGCTGCAGTTATTCATTCC3′	1137–1743	606	*L. ivanovii*, *L. seeligeri*
IvaSee-R1	5′ATCTAAGAATTTTTGTTTTAGT3′			
Wel-F1	5′TTCTCGTATTATCGGTTTACCA3′	2344–2581	237	*L. welshimeri*
Wel-R1	5′GCTTCAAGATAGATTTCTTTCAA3′			
Mar-F1	5′AGAATATATTTGGAACAGCATC3′	246–2059	1813	*L. marthii*
Mar-R1	5′GTTCGATTGCACGGATGGAAAG3′			

*^a^* Underlining indicates artificial nucleotide addition sites; translation start codon is indicated in bold.

## Supplementary Materials

Supplementary File 1
