# Supplementary material for: Novel PCR Assays Complement Laser Biosensor-Based Method and Facilitate Listeria Species Detection from Food
Source: Sensors (Basel). 2015 Sep 8;15(9):22672–91. doi: 10.3390/s150922672 (PMC4610479; doi:10.3390/s150922672)
Supplement: Supplementary File 1 [file sensors-15-22672-s001.pdf]

OPEN ACCESS

sensors

ISSN 1424-8220

www.mdpi.com/journal/sensors

Supplementary Information

Novel PCR Assays Complement Laser Biosensor-Based Method and Facilitate *Listeria* Species Detection from Food. *Sensors* 2015, *15*, 22672-22691

Kwang-Pyo Kim [1,2,†], Atul K. Singh [1,†], Xingjian Bai [1], Lena Leprun [1,‡] and Arun K. Bhunia [1,3,*]

1 Molecular Food Microbiology Laboratory, Department of Food Science, Purdue University, West Lafayette, IN 47907, USA; E-Mails: kpkim@jbnu.ac.kr (K.-P.K.); aksingh@purdue.edu (A.K.S.); bai16@purdue.edu (X.B.); l.leprun@laposte.net (L.L.)

2 Department of Food Science and Technology, College of Agriculture and Life Sciences, Chonbuk National University, Jeonbuk 561756, Korea

3 Department of Comparative Pathobiology, Purdue University, West Lafayette, IN 47907, USA

‡ Present address: CROUS de Dijon, Dijon Cedex 21012, France.

† These authors contributed equally to this study.

* Author to whom correspondence should be addressed; E-Mail: bhunia@purdue.edu; Tel.: +1-765-494-5443; Fax: +1-765-494-7953.

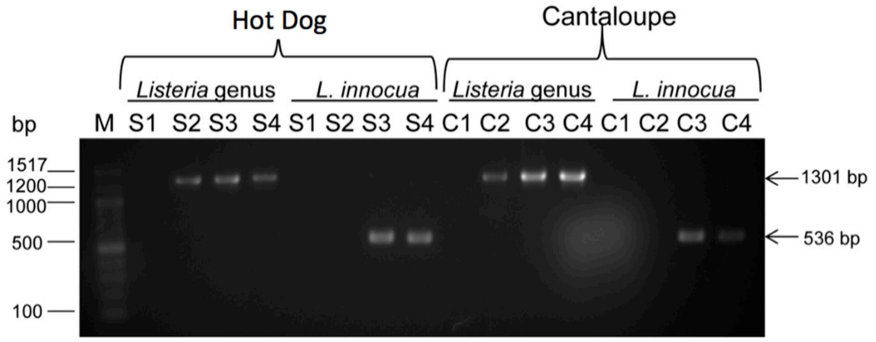

Figure S1. Application of *lap* gene-based *Listeria sensu stricto* specific primers (ELAP-F/LIS-R1) and 460 *Listeria innocua* (InnF1/InnR1)-specific primer sets for detection of *L. monocytogenes* and *L. innocua* in food system (hotdog sample, S and Cantaloupe, C). Uninoculated sample, S1/C1; sample inoculated with *L. monocytogenes* F4244, S2/C2; sample inoculated with *L. innocua* F4248, S3/C3; and sample inoculated with *L. monocytogenes* F4244 and *L. innocua* F4248, S4/C4. To detect *Listeria* genus and *L. innocua*, primer set ELAP-F1/LIS-R1 and Inn-F1/Inn-R1 were used in the PCR amplification for DNA extracted from enriched sample. M, 100 bp DNA ladder (NEB, Ipswich, MA).

Table S1. List of bacterial culture and specificity of *lap* gene-specific primer sets used in this study.

Isolates (serotype) [a]	Ribotype [b]	PCR Amplification with Primer Combinations			
		ELAP-F1/LIS-R1	Inn-F1/Inn-R1	IvaSee-F1/IvaSee-R1	Wel-F1/Wel-R1
Listeria strains					
L. monocytogenes ATCC15313 (1/2a)	DUP-1042	+	−	−	−
L. monocytogenes Scott A (4b)	DUP-1042	+	−	−	−
L. monocytogenes F4244(4b)	DUP-1044	+	−	−	−
L. monocytogenes ATCC35152 (1/2a)	DUP-1030	+	−	−	−
L. monocytogenes ATCC43257 (4b)	NT	+	−	−	−
L. monocytogenes ATCC19118 (4e)	DUP-1038	+	−	−	−
L. monocytogenes ATCC7644 (1/2c)	NT	+	−	−	−
L. monocytogenes ATCC19114 (4a)	DUP-1059	+	−	−	−
L. monocytogenes ATCC2540 (3b)	DUP-1052	+	−	−	−
L. monocytogenes ATCC19116 (4c)	DUP-1061	+	−	−	−
L. monocytogenes ATCC19117 (4d)	DUP-1042	+	−	−	−
L. monocytogenes ATCC19112 (1/2c)	DUP-1039	+	−	−	−
L. monocytogenes ATCC19115 (4b)	DUP-1042	+	−	−	−
L. ivanovii SE98	NT	+	−	+	−
L. ivanovii ATCC19119	DUP-1021	+	−	+	−
L. ivanovii V12 (5)	NT	+	−	+	−
L. ivanovii V35 (5)	NT	+	−	+	−
L. ivanovii V119	NT	+	−	+	−
L. ivanovii LM6	NT	+	−	+	−
L. ivanovii SLCC4769	NT	+	−	+	−
L. ivanovii V195 (5)	NT	+	−	+	−
L. ivanovii LA29	NT	+	−	+	−
L. ivanovii SE136	DUP-1021	+	−	+	−
L. ivanovii 2732 NVSL	NT	+	−	+	−
L. ivanovii 2875 NVSL	NT	+	−	+	−
L. innocua F4248	DUP-1006	+	+	−	−
L. innocua V57 (6a)	DUP-1005	+	+	−	−
L. innocua V58 (6b)	DUP-1009	+	+	−	−
L. innocua V11 (6a)	DUP-1009	+	+	−	−
L. innocua F4247	DUP-1018	+	+	−	−
L. innocua V22 (6a)	DUP-1005	+	+	−	−
L. innocua V24 (6b)	DUP-1005	+	+	−	−
L. innocua ATCC33090 (6a)	DUP-1009	+	+	−	−
L. innocua C91-2(L)	DUP-1005	+	+	−	−
L. innocua LA-1	DUP-1009	+	+	−	−
L. seeligeri SE31	NT	+	−	+	−
L. seeligeri SLCC3954	NT	+	−	+	−
L. seeligeri V45 (1/2b)	NT	+	−	+	−
L. seeligeri V34 (1/2b)	NT	+	−	+	−
L. seeligeri V13 (1/2b)	NT	+	−	+	−

Table S1. *Cont.*

Isolates (serotype) [a]	Ribotype [b]	PCR Amplification with Primer Combinations			
		ELAP-F1/LIS-R1	Inn-F1/Inn-R1	IvaSee-F1/IvaSee-R1	Wel-F1/Wel-R1
L. welshimeri ATCC35897	NT	+	−	−	+
L. welshimeri LM156	NT	+	−	−	+
L. welshimeri 105_2(2L)	NT	+	−	−	+
L. grayi LM37	NT	−	−	−	−
L. grayi ATCC19120	NT	−	−	−	−
L. marthii BAA-1595	NT	+	−	NT	NT
L. rocourtiae CIP109804	NT	−	−	NT	NT
Non-Listeria cultures					
Enterobacter aerogenes	NT	−	−	−	−
Serratia marcescens	NT	−	−	−	−
Hafnia alvei	NT	−	−	−	−
Lactobacillus casei	NT	−	−	−	−
Lactobacillus acidophilus NRRL B131910	NT	−	−	−	−
Bacillus cereus ATCC3432	DUP-6078	−	−	−	−
Escherichia coli EDL933 (O157:H7)	DUP-3064	−	−	−	−
Salmonella enterica serovar Typhimurium ATCC13096	NT	−	−	−	−

[a] Total strain 55 bacterial strains were used in this study, comprising of 47 strains of *Listeria* specis (*L. monocytogenes*, *n* = 13; *L. ivanovii*, *n* = 12; *L. innocua*, *n* = 10; *L. seeligeri*, *n* = 5; *L. welshimeri*, *n* = 3; *L. grayi*, *n* = 2; *L. marthii*, *n* = 1; *L. rocourtiae*, *n* = 1); Non-*Listeria* strains, *n* = 8; NT, not tested; [b] Ribotyping was performed in an automated RiboPrinter (Qualicon, Inc.) with the *Eco*RI restriction enzyme (Gray *et al*., 2005). Ribopatterns were compared with the RiboPrinter database for culture identification.

Table S2. Cross validation matrix obtained after image analysis of the scatter of eight Listeria species colonies grown on brain-heart infusion (BHI) agar medium, related to Figure 4.

Listeria species[a]	*L. monocytogenes*	*L. innocua*	*L. grayi*	*L. seeligeri*	*L. marthii*	*L. weslhimeri*	*L. rocourtiae*	*L. ivanovii*
L. monocytogenes	**97.7**	0	0	0	0	0	2.3	0
L. innocua	0	**100**	0	0	0	0	0	0
L. grayi	0	0	**66.4**	1.2	0	5.4	0.3	26.7
L. seeligeri	0	0	1.8	**94.9**	0	3.3	0	0
L. marthii	1.7	0	1.2	0	**95.2**	0	0	2
L. weslhimeri	0	0	6.6	2.9	0	**88.9**	0	1.6
L. rocourtiae	0	0	0	0	0	0	**100**	0
L. ivanovii	0	0	45.3	1.3	0	3.2	0	**50.1**

[a] Cross-validation matrix was generated with image classifier after analysis of around 80 scatter pattern per species.

© 2015 by the authors; licensee MDPI, Basel, Switzerland. This article is an open access article distributed under the terms and conditions of the Creative Commons Attribution license (http://creativecommons.org/licenses/by/4.0/).
